# Envisioning the future of clinical analytics: a modified Delphi process in New South Wales, Australia

**DOI:** 10.1186/s12911-020-01226-7

**Published:** 2020-09-04

**Authors:** Kim Sutherland, Wilson Yeung, Yoke Mak, Jean-Frederic Levesque, the NSW Health Clinical Analytics Working Group

**Affiliations:** 1 NSW Agency for Clinical Innovation, Chatswood, NSW Australia; 2 eHealth NSW, Chatswood, NSW Australia; 3 Centre for Primary Health Care and Equity, UNSW, Randwick, New South Wales Australia

**Keywords:** Clinical analytics, Visioning, Consensus, Delphi

## Abstract

**Background:**

Clinical analytics is a rapidly developing area of informatics and knowledge mobilisation which has huge potential to improve healthcare in the future. It is widely acknowledged to be a powerful mediator of clinical decision making, patient-centred care and organisational learning. As a result, healthcare systems require a strategic foundation for clinical analytics that is sufficiently directional to support meaningful change while flexible enough to allow for iteration and responsiveness to context as change occurs.

**Methods:**

In New South Wales, the most populous state in Australia, the Clinical Analytics Working Group was charged with developing a five-year vision for the public health system. A modified Delphi process was undertaken to elicit expert views and to reach a consensus. The process included a combination of face-to-face workshops, traditional Delphi voting via email, and innovative, real-time iteration between text re-formulation and voting until consensus was reached. The six stage process engaged 35 experts — practising clinicians, patients and consumers, managers, policymakers, data scientists and academics.

**Results:**

The process resulted in the production of 135 ideas that were subsequently synthesised into 23 agreed statements and encapsulated in a single page (456 word) narrative.

**Conclusion:**

The visioning process highlighted three key perspectives (clinicians, patients and managers) and the need for synchronous (during the clinical encounter) and asynchronous (outside the clinical encounter) clinical decision support and reflective practice tools; the use of new and multiple data sources and communication formats; and the role of research and education.

## Background

New approaches to clinical analytics have the potential to transform healthcare delivery by reshaping clinical decision-making practices, influencing patient-provider interactions, altering patient trajectories and outcomes, and driving improvements in quality of care [1–3]. Realising this potential is not straightforward however, and requires strategic decisions about feasibility; preferred approaches to clinical and organisational transformation; and appropriate investment in information collection, digital technology and change management processes. In complex adaptive systems such as health where there is a vast array of options, priorities and perspectives, these types of strategic decisions are best guided by a shared and well informed vision of the future [4].

Many developed healthcare systems are seeking to capitalise on the promise of digital healthcare [4–7] and more specifically clinical analytics [8]. In New South Wales (NSW), the most populous state in Australia, eHealth and data analytics are a key strategic priority [9].

The Clinical Analytics Working Group is a sub-committee of the NSW Health Analytics Steering Committee. The working group’s role is to advise on how analytics can be harnessed to support clinical and organisational decision making, leading to improved patient safety, quality and outcomes. It seeks to find solutions that will provide timely access to electronically captured data from clinical systems and other relevant sources; and transform it into meaningful information to better inform service delivery planning for effective and efficient clinical care delivery. One of the key tasks assigned to the working group is to develop and articulate a five-year vision for clinical analytics for NSW Health. This paper describes how, through a series of workshops and a modified Delphi process, this has been achieved.

### What is meant by *clinical analytics*?

There is considerable ambiguity in the terminology used regarding clinical analytics – with terms such as analyses, analytics, health analytics, data analytics - used interchangeably but often with different meaning.

In this paper and in the Clinical Analytics Working Group, the term, *clinical* is used to denote the examination and treatment of patients – it refers to patient-provider interactions and spans preventive, diagnostic, therapeutic and supportive care*. Data analytics* relates to automated processes that produce information from raw data. As a concept, it is distinct from *data analysis* which is purposeful and sometimes iterative interrogation of data to produce information [10]. *Clinical analytics* relates to the automated processes that produce clinically-relevant information to support clinician-mediated decisions, patient-mediated decisions and shared decisions [11]. Clinical analytics utilises data analytics and data analyses.

### Clinical analytics and clinical decision support

Clinical decision support represents one of the most powerful applications of clinical analytics, and aims to make data about a patient easier to access or more apparent; and to foster problem solving and guide action by users. Defined as “the use of information and communication technologies to bring relevant knowledge to bear on the healthcare and wellbeing of a patient,” users of clinical decision support include doctors, nurses, scientists and technical staff, allied health professionals, pharmacists, patients and carers ([10]: p8).

**Fig. 1:**
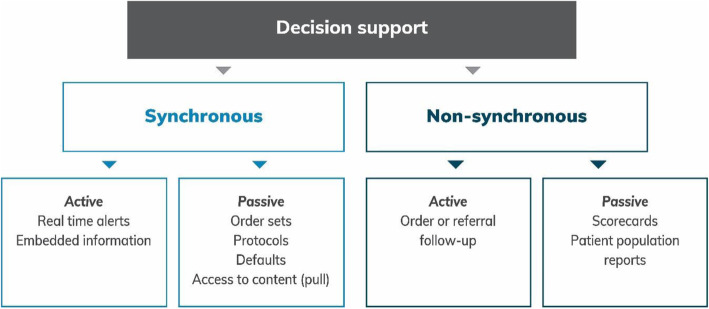
Synchronous and asynchronous decision support. Source: Adapted from Sanders [12]

Clinical decision support encompasses two main types of information flow - synchronous and asynchronous – each of which can be active or passive in nature [12] (Fig. 1).

Synchronous clinical decision support most frequently provides standardised information, triggered by a particular parameter such as a pharmacy order or a set of laboratory results and in the form of real-time pop ups, dialogue boxes and advice. When provided in an alert format, this type of decision support can be disruptive of workflow and experience to date has shown that a high proportion of alerts are ignored or overridden [13, 14].

Asynchronous clinical decision support provides feedback after an index decision is made. It provides opportunities for aggregation and comparisons with other providers, as well as time series analyses. It can provide a trigger for reflective practice and opportunities to consider and possibly reset heuristics.

### Why a five-year vision?

The articulation of a five-year vision is needed to harness the potential of clinical analytics in improving healthcare - supporting clinical decision making, patient-centred care and organisational learning. The objectives for the process are to build a shared understanding of the potential opportunities of clinical analytics; unearth underlying values and understanding held by stakeholders which can be leveraged to coordinate and guide improvement efforts in an integrated way; and highlight common barriers and levers for change.

The Working Group agreed on two key parameters for the vision at the outset of the process. First, that a tripartite perspective was needed - giving consideration to the potential benefits and implications for clinicians, patients and managers. Second, that the five-year time horizon should encapsulate projects or initiatives that could be completed in that time frame; as well as more ambitious projects that will be developed and piloted in the 5 year period, but require an extended implementation period.

### Method - building consensus through a modified Delphi process

A Delphi method is an iterative process in which a group of expert stakeholders come to a structured consensus view on a particular topic [15]. Delphi processes have been successfully conducted to establish research priorities for a range of different topic areas such as identifying challenges for coordination in healthcare services [16], determining the future organisation of thrombectomy services in England [17], agreeing research priorities for patient safety in mental health [18] and for emergency medicine physicians in Australia and New Zealand [19].

The Delphi method, originally developed in the 1950s, entails engaging a group of experts - generally through a number of rounds where statements or options for the issue under consideration are circulated; feedback is elicited, collated and analysed; and the resulting syntheses or views are re-circulated to the group for further refinement and ratification [20]. The goal is to reduce the range of responses and arrive at something close to expert consensus.

In the classic Delphi process there is no interaction between experts and researchers. We undertook a modified Delphi which incorporated a face to face workshop, an online survey and feedback, and real-time iteration between anonymised redrafting of text and voting using Sli.do software. Such modifications are frequently used to more fully explore context, enablers and barriers for change [21–23]. These processes sought first to elicit the breadth of experts’ views of the future; and second to establish a convergence of opinion of a likely five-year vision. As a secondary outcome, the process also highlighted consistently identified barriers and levers for change.

**Fig. 2:**
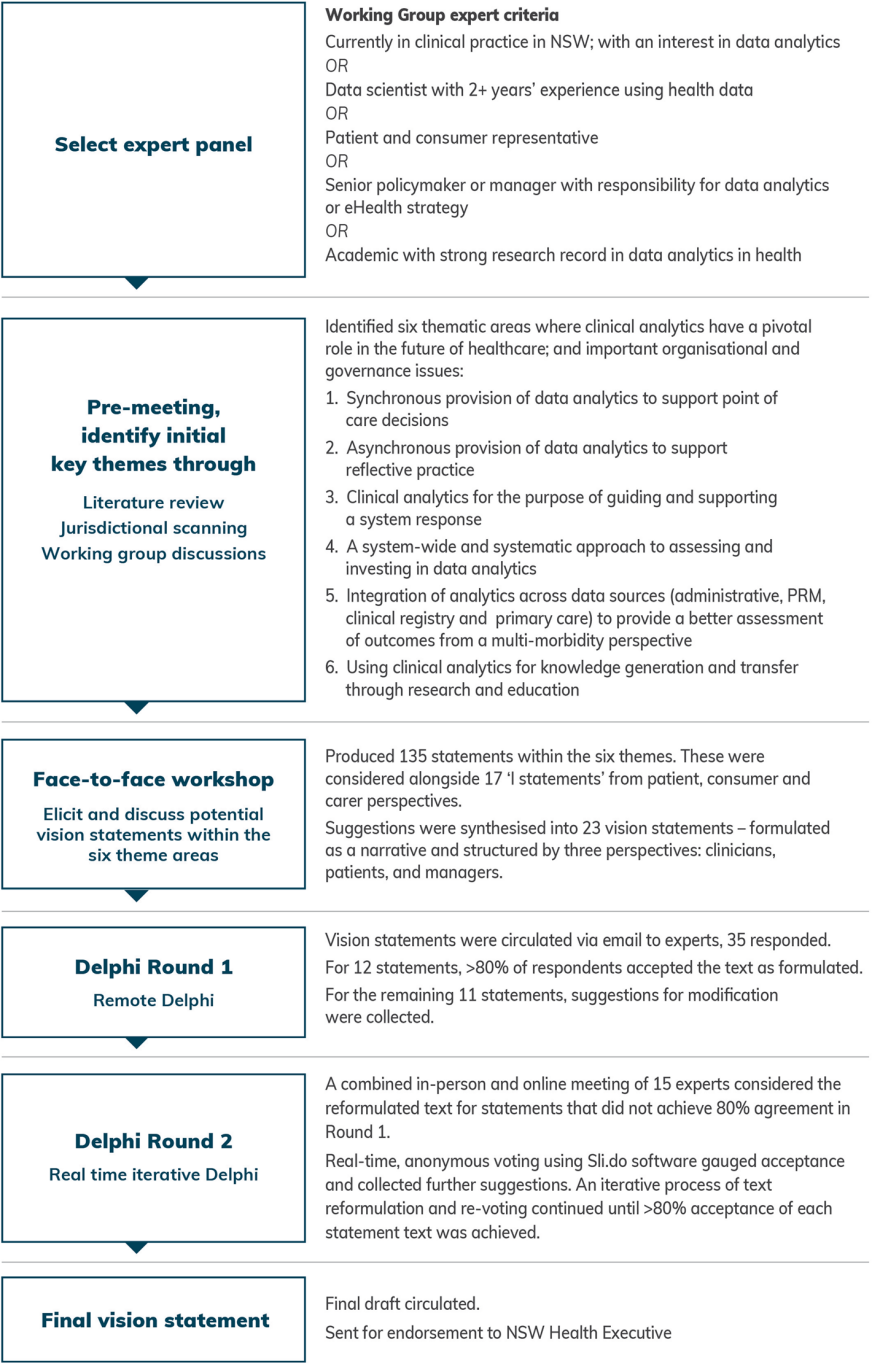
Schematic of the process used to develop a five-year vision for clinical analytics in NSW

The process used to develop the five-year vision for clinical analytics is summarised in Fig. 2.

#### Six stage process to develop the vision

##### Stage 1: Identify expert panel

The Working Group agreed that experts would be eligible for inclusion in the Delphi process if they were: active in NSW as: a practising clinician, with an interest in data analytics; a data scientist with 2+ years’ experience using health data; a patient and / or consumer representative with an interest in health data; a senior policymaker or manager with responsibility for data analytics or eHealth strategy or; an academic with strong research record in data analytics in health.

In August 2018, invitations were sent by email to 35 experts; and 32 agreed to participate in the visioning exercise (91% response rate). The characteristics of the groups are summarised in Table 1.

**Table 1:** Job titles of experts who participated in developing the five-year vision

Job titles of participants in the five-year vision exercise		
Chief Nursing Information Officer (M) Director of Performance (M) Clinical Advisor (C/D) Chief Nursing Information Officer (M) Postdoctoral Research Fellow (D) Neurologist (C) Associate Professor Cardiologist (C) Director Performance Reports (M) Senior Project Officer (M) Chief Medical Information Officer (C/D) Chief Executive (M)	Registrar, Royal Australasian College of Medical Administrators (RACMA) (M) Director Biomedical and Health Informatics (D) Director of Clinical Governance & Information Services (M) Specialist Advisor (D/M) Director Data & Analytics Portfolio (D/M) Clinical Risk & Practice Improvement Manager (M) Strategic Advice & Design Governance Lead (M) Clinical Director (C/M) Director of Medical Services (M)	Medical Advisor (C/M) Manager Health Analytics Business Support (D) Executive Director Clinical Governance (M) GP Specialist Consultant (C) Executive Director (M) Biostatistician (D) Service Rationalisation Project Manager (M) A/Director Allied Health (C/M) Director of Medical Services (M) Rural Director of Medical Services (C/M) Implementation Manager (M)

*C* Clinician, *D* Data Scientist, *M* Manager

##### Stage 2: Pre-meeting identification of thematic areas

An initial set of thematic areas was generated from Working Group deliberations, and supplemented by a rapid review of peer reviewed and grey literature. Search terms were “clinical analytics”; “horizon scanning”; “data analytics”. Results from PubMed searches were:

Search ((“strategic vision” AND “last five years”[PDat))) OR “horizon scan” 153 items found.

Search ((“data analytics” [Title/Abstract]) OR (“clinical analy*”[Title/Abstract]) OR “strategic vision” OR “horizon scan” AND “last five years”[PDat])) 117 items found.

Articles were screened for key topics to be used as catalysts for discussion and consideration. Data were extracted into a thematic table.

Six key areas of healthcare where clinical analytics could have a pivotal role in the future were identified. They were:

1. The synchronous provision of data analytics to support point of care decisions
2. Asynchronous provision of data analytics to support reflective practice
3. Clinical analytics for the purpose of guiding and supporting a system response
4. A system-wide and systematic approach to assessing and investing in data analytics
5. Integration of analytics across data sources (administrative, patient reported measures, clinical registry and primary care) to provide a better assessment of outcomes from a multimorbidity perspective
6. Using clinical analytics for knowledge generation and transfer through research and education

##### Stage 3: Face to face workshop

A workshop in September 2018 brought together 32 experts, and was run by expert facilitators from the implementation team of the NSW Agency for Clinical Innovation. Data were collected through physical artefacts; video; and contemporaneous note taking.

Participants were asked to perform three key tasks. First, to articulate what they considered to be key elements of the 5 year vision in each of the six thematic areas. They were asked to consider what could be accomplished within three time periods: achieved in 5 years’ time; pilots in 5 years’ time; either initiatives in train OR strategy / plans in place in 5 years’ time.

The second key task was to consider a set of ‘I statements’ that addressed clinical analytics from a patient, consumer and carer perspective (Appendix 1). These statements were generated by England’s National Voices organisation – which draws on 160 health and social care charities – covering a diverse range of health conditions and communities, and connecting with the experiences of millions of people.

For the third key task, participants were asked to identify key barriers and enablers to achieving the vision for clinical analytics.

##### Stage 4: Delphi round 1

Two researchers collated and independently clustered the themes. Results were compared and any discrepancies resolved by discussion. This resulted in 23 statements, structured into clinician, patient and manager perspectives. These vision statements were circulated via email to experts, 35 responded. Experts were asked to select from the following options:

- I accept this statement as formulated
- I do not accept this statement
- I propose a modification to the statement – change it to _____.

For 12 statements, > 80% of respondents accepted the text as formulated. These statements were incorporated into the final vision narrative. For the remaining 13 statements, suggestions for modification were collected.

##### Stage 5: Delphi round 2

A combined in-person and online meeting used real-time voting and redrafting processes via the Sli.do software. For each of the 13 statements to be considered, the original text was shown, the suggested modifications and a draft reformulation was reviewed. A round of anonymised voting ascertained whether consensus had been reached. If not, further redrafting occurred followed by another voting round. This iterative process continued until > 80% agreement was achieved.

##### Stage 6: Circulate final narrative statement

The final agreed narrative was circulated to all participants for final endorsement.

## Results

The workshop generated 135 statements across the six thematic areas. These statements were parsed into vision narratives within each of the stakeholder perspectives: clinician (C1-C6); patients (P1 –P3); and managers (M1 – M3) (see Appendix 2 for the statements, mapped to thematic areas and vision narratives).

During the face to face workshop, a sticker voting system was used to prioritise the statements and those with more than 10 votes are shown in Table 2.

**Table 2:** The clinical analytics vision statements accorded highest priority by workshop participants

Vision statement	Number of votes
Integration of data analytics with Electronic Medical Record (EMR)	14
Streamlined system-level reporting or a reporting framework for variation in clinical care, patient journeys and outcomes	13
All clinicians will receive regular data about their service delivery and outcomes from the previous quarter and with time trends analyses. These data will be discussed within clinical teams so that clinicians can collectively assess the data and identify causes of variation and plan improvements	12
There is a robust mechanism and framework to identify, prioritise and support the introduction of system wide clinical analytic initiatives	12
Real time monitoring, predicting, alerting and evaluating care for patient safety (e.g. deteriorating patients)	11

One session of the face to face workshop elicited barriers and enablers. The results are tabulated in Appendix 3.

In the initial Delphi round conducted by email, the percentage of respondents who accepted the statements as formulated ranged from 55 to 100%. Of the 23 statements circulated 12 achieved > 80% agreement (Appendix 4). The remaining 13 statements proceeded to the next Delphi round.

The second Delphi round was a meeting with in-person and online participants who had their responses collected anonymously. The 90 min session saw all statements reach agreement, taking between 1 to 5 iterations (Appendix 5). No statements were rejected outright, and all were modified until consensus was reached. The final result of the visioning exercise was synthesised into a one-page narrative (Table 3).

**Table 3:** The five-year vision for clinical analytics in NSW – a one-page narrative

In five years’ time … Clinicians will use patient reported measures as a part of routine care. The measures will be used for diagnosis, prognosis and clinical decision making. Clinically validated algorithms will assess case histories, diagnoses and risk profiles; and will facilitate safe and effective clinical care. Targeted and well validated alerts will highlight risk and safety issues. Aggregated, time-series data will be collected unobtrusively through the electronic medical record (eMR) and routine clinical tasks. Clinicians will have access to relevant and timely information that highlights any unwarranted clinical variation and supports reflective and current best practice. Information will be available at the point of care on concordance of clinicians’ care with evidence-based practice; risk adjusted patient outcomes; benchmarking and peer comparisons; time-series and patient trajectories. Advanced analytics or artificial intelligence (AI) approaches will be deployed to discern novel patterns in complex and large datasets and guide the development of algorithms. Analytics-driven clinical audit processes will draw on “virtual registries” to personalise learning. Feedback will be informed by the evidence on clinical decision making – incorporating passive ‘automated’ predictive analytics as well as peer to peer and expert feedback. Data will be discussed within clinical teams so that clinicians can collectively assess the data and identify causes of variation and plan improvements. Clinical research will be informed by timely and efficient access to linked data, big data, “virtual registries” and analytics. Efforts will be underway to secure wider data linkage to incorporate non-health sources. Clinician training will incorporate the use of analytics and address issues such as managing risk and uncertainty. Patients will be assured that their data are appropriately secure and used to support clinical care and quality improvement. They will be firmly established as key informants in healthcare – providing data about their health status, experience and outcomes. Patients who chose to, will be engaged in monitoring their health using technologies that can communicate with information systems. Patients will be enabled and supported to access their own data and to use it to manage their health. With their consent, patient self-management will be prompted by algorithm enabled alerts. Managers will be confident that monitoring and measurement systems are reliably and sensitively assessing healthcare services. They will be able to test models of reconfiguration and structural changes using data analytics. Real time alerts regarding impending surges in demand in acute care areas such as emergency departments, operating theatres and critical care units will be used to manage workflows, staffing and bed management. Service level and system managers will utilise data from clinical analytics alongside administrative and other data to guide policy development and improve performance. There will be a robust mechanism and framework to identify, prioritise and support the introduction of system wide clinical analytic initiatives.	

## Discussion

Predicting the future is a fraught task. Doing so in areas technology plays a pivotal role is particularly challenging. In 1994, the view of an official in the UK’s Whitehall was that “email will never catch on” [24] – highlighting the danger of relying on a singular perspective in envisioning the future.

Our intention in developing a shared view of the future for clinical analytics is not to formulate a precise prediction of the future. Our goal is to draw on a range of perspectives and expertise so that NSW Health can be positioned for a range of future scenarios and enhance its ability to respond to changing needs, policy priorities and circumstances.

The modified Delphi process resulted in a concise picture of what we expect the clinical analytics landscape to look like in 2024. It also revealed a number of enablers and barriers that will affect the realisation of the vision.

### Enablers and barriers

Deliberations about how to achieve the five-year vision focused on the need for frameworks, collaborative working and a high regard for patients’ perspectives. Experts emphasised the importance of collaboration in clinical analytics approaches between clinicians, and analysts and data specialists; the breaking down of silos. At the same time, they acknowledged the importance of delivering on patients’ expectation that their data are used to drive improvement. A key enabler was seen to be a framework that captures the principles and real world practice of clinical decision making – to act as a guide for the type of analyses, alerts; and reporting that will secure behaviour change and quality and safety improvement; and will minimise cognitive load on clinicians.

There was a shared imperative to be ‘realistic’ in expectations, enthusiasm for the Pareto principle or ‘80–20’ rule – emphasising that the development of clinical analytics should be guided by a ‘satisficing’ approach [25] hat does not seek perfection nor complete accuracy. Sandbox initiatives and capacity for experimentation and experiential learning about potential solutions and ‘what works’ were regarded to be highly desirable.

### Reflecting on clinical decision support

The modified Delphi process placed significant focus on how clinical analytics can support clinical decision making and the deliberations of the group prompted us to develop a conceptual model that considers four key areas (Table 4) [26].

**Table 4:** The potential role of clinical analytics in clinical decision support of the future

	Types of information about the patient as an individual	Types of information about the patient as part of a cohort
**Synchronous** **(At the time of the clinical encounter)**	“This patient has incongruent patient-reported-measures (PRM) and lab test results” “This patient’s clinical parameters suggest he or she is at high risk of an adverse event” “This patient’s PRMs have been trending downwards for the past six months”	“This patient is in the highest frailty decile of your patient cohort” “This patient’s recent trajectory has diverged from that of his or her cohort”
**Asynchronous** **(At a time outside the clinical encounter)**	“A patient you saw last month has experienced significant changes in his or her clinical parameters” “The treatment you prescribed for this patient resulted in a change in trajectory”	“More than 50% of your patients are clustered in the bottom 10% of the cohort” “The treatments you prescribe for patients with back pain differ significantly from your peers and outcomes are worse”

### Limitations

There are several limitations with this study. First, in terms of expert recruitment, our Delphi panel was founded on a pre-existing group that had been convened to advise NSW Health on clinical analytics. While we supplemented this group, patients were under-represented. Second, the expert panel varied in size and composition over the three rounds of activity. For the idea generation phase, the core group was supplemented with targeted invitations to bridge potential gaps in coverage of key stakeholders, interest groups and knowledge. For the Delphi rounds, participation rates varied with 53% of the core group voting in all rounds and several substitutions of organisational representatives between the rounds. Third, for many of the vision statements we did not reach 100% consensus across the group. Given the nature of the question and the call for speculation about the future inherent in the process, we agreed that 80% agreement would provide sufficient precision to allow us to conclude that this was a shared vision. Future work aims to overcome the limitation of under-representation of some groups – through broader engagement through crowdsourcing techniques.

## Conclusion

Clinically led improvement, enabled by new technology and analytic capacity, is transforming the delivery of healthcare and our management of population health. Yet strategic decisions about the scale of clinical transformation and associated investment in information, analytics, and digital technology are often reactive, destined to play catch up and make do [4].

Perhaps this reticence is based on the assumption that predicting the future is something of a fool’s errand. Yet forethought and foresight are absolutely essential if we are to harness to potential of innovation in healthcare. The trick is to be sufficiently prescient to be prepared for both expected and unexpected gains and consequences of profound change.

## Supplementary information

**Additional file 1: Appendix 1.** Reflecting on I Statement. **Appendix 2.** Clinical analytics fiver-year statements (original thematic areas and parsed to final narrative one-pager). **Appendix 3.** Barriers and enabler identified in workshop. **Appendix 4.** Online Survey. **Appendix 5.** Final Delphi

## Data Availability

All data are provided in Appendices.

## Abbreviations

EMR: Electronic Medical Record
NSW: New South Wales
PRM: Patient Reported Measures
