# Supplementary material for: Envisioning the future of clinical analytics: a modified Delphi process in New South Wales, Australia
Source: BMC Med Inform Decis Mak. 2020 Sep 4;20:210. doi: 10.1186/s12911-020-01226-7 (PMC7650225; doi:10.1186/s12911-020-01226-7)
Supplement: Supplementary file 1 — Additional file 1: Appendix 1. Reflecting on I Statement. Appendix 2. Clinical analytics fiver-year statements (original thematic areas and parsed to final narrative one-pager). Appendix 3. Barriers and enabler identified in workshop. Appendix 4. Online Survey. Appendix 5. Final Delphi [file 12911_2020_1226_MOESM1_ESM.docx]

**SUPPLEMENTARY MATERIAL**

**Appendix 1: Reflecting on I statements –**

The development of:

*My care is consistently delivered to a high standard*

*The system finds me and intervenes at an early stage to avoid a crisis*

*I have the information and support I need to manage my condition*

*I know that professionals involved in my care will seek out the information they need to help me understand the risks and benefits of treatments, in the context of me, my situation and how I manage my health.*

*I know that all of the potential risks and benefits of accessing a treatment will be clearly explained.*

*When I interact with the system it is consistent and I have trust in my care*

*I know that professionals involved in my care will ensure that they have up-to-date knowledge and information about all the treatments that may be available to me and will provide me with the opportunity to find out about this*

*I know that professionals involved in my care have available, and use, information about how their practice compares to others*

*The professionals involved in my care work as a team and are able to draw on all the relevant information about my care*

*I (and those that support me) can access the specialist advice I need wherever and whenever I need it*

*Whenever I use a service, there are not unnecessary tests, delays or wasted visits*

*I know that the services that support me are always trying to find ways to improve my experience and the outcomes that are important to me*

*I know that people with a wide range of characteristic and backgrounds will be included in and benefit from developments in data analytics*

*I know that patients and citizens are involved in deciding priorities for data analytics*

*I know that what matters most to patients and citizens is taken into account in setting priorities for data analytics*

*I get accessible information and support that helps me understand how priorities are set*

*I get accessible information and support that helps me to understand how I can get involved in decision making about data analytics*

Adapted from: National Voices, 2016 <https://www.nationalvoices.org.uk/sites/default/files/public/publications/involving_patients_and_service_users_-_i_statements_for_research_and_innovation_oct_2016_0.pdf>

**Appendix 2: Clinical analytics five-year vision statements (original thematic areas and parsed to final narrative one pager)**

| **Statement** | **Themes** | **Narrative statements** |
| --- | --- | --- |
| **Clinicians’ Perspective** | | |
| C1: Clinicians in five years’ time will, as a matter of course, use patient reported measures (gathered via patients’ phones and tablets), incorporated into other diagnostic and prognostic markers and inputs. Algorithms will assess patterns, patient trajectories and risk strata for patients and this information will be considered in the consultation. Very targeted and well validated alerts will highlight risk and safety issues. | | |
| Real time monitoring, predicting, alerting and evaluating care for patient safety (e.g. deteriorating patients) and address unwarranted clinical variation | 1 | C1 |
| Contemporaneous patient-reported symptom and quality of life metrics available at all consultations | 1 | C1 |
| Patient-clinician consultations will be informed by predictive analytics | 1 | C1 |
| Electronic PROMS capture is routine in acute and outpatient care settings | 1 | C1 |
| Live clinical data capture | 1 | C1 |
| Analytics of trends in patient-reported outcomes are available for the purpose of tailoring therapeutic approaches contributing to personalised medicine | 1 | C1 |
| Real time monitoring, predicting, alerting and evaluating care for patient safety (e.g. deteriorating patients) | 1 | C1 |
| Clinical decision support integrated and supported as mainstream by clinicians | 1 | C1 |
| Real-time medical management with genomics | 1 | C1 |
| Multimorbidity and personalised medicine | 5 | C1 |
| Exemplars of connecting big data and small data at point of care | 4 | C1 |
| C2: Aggregated, time-series data will be collected unobtrusively through eMR and other routine clinical tasks. Clinicians will have access to timely information that allows for reflective practice, highlighting whether their results point to unwarranted clinical variation – that is, as “patient care that differs in ways that are not a direct and proportionate response to available evidence; or to the healthcare needs and informed choices of patients”. Information will be automatically generated on:   - Concordance with evidence-based practice - Diffusion rates of new technologies - Risk adjusted patient outcomes - Benchmarking comparisons - Time-series and patient trajectories | | |
| Integration of data analytics with EMR | 1 | C2 |
| Streamlined system-level reporting or a reporting framework for variation in clinical care, patient journeys and outcomes | 3 | C2/M1 |
| All clinicians will receive regular data about their service delivery and outcomes from the previous quarter and with time trends analyses. These data will be discussed within clinical teams so that clinicians can collectively assess the data and identify causes of variation and plan improvements. | 2 | C2 |
| Data analytics-based decision-support tools are routinely used | 1 | C2 |
| Real-time reporting approaches based on decision making theory are in place | 1 | C2 |
| Aggregated, time series and comparative reporting is integrated with the EMR | 2 | C2 |
| There is the capacity to build bespoke analyses and cohorts e.g. to build insight about complex patients | 2 | C2 |
| Clear and useful data visualisation tools (designed by clinicians) are available for clinicians, patients and policymakers | 2 | C2 |
| Integrates predictors of outcome utilising known evidence-based pathways / scores | 1 | C2 |
| Focus on process as much as outcome | 5 | C2 |
| Clinical guidelines and clinical pathways developed and evaluated / monitored using clinical data analytics | 4 | C2 |
| Regular standardised data reports or access to (push); standardised data definitions (QIDS) | 2 | C2 |
| Ability to pull data sets of interest | 2 | C2 |
| Enhanced research-based information is available - spanning knowledge about implementation of clinical analytics, validation, new insights emerging from clinical analytics, value, unintended consequences | 6 | C2 |
| Pathology decision support at the bedside | 1 | C2 |
| C3: Machine learning processes, deployed to discern novel patterns in complex and large datasets, will inform the development of algorithms and highly specific alerts. | | |
| Machine-learning /AI based alerting systems that informs real time clinical decision making | 1 | C3 |
| New data linkages and triangulation of data incorporated into clinical analytics | 1 | C3 |
| Triangulated data from multiple sources e.g. clinical and corporate data / patient experience / survey data including workforce | 2 | C3 |
| Algorithms for stepwise approach to quality of care and safety monitoring - unobtrusive surveillance of multiple metrics and tailored alerts for metrics which require action / attention | 2 | C3 |
| Enhanced predictive risk analytics using machine learning /AI to identify patients at high risk of hospitalisation or other QOL outcomes | 5 | C3 |
| Peer benchmarking and test ordering as an example of real-time decision support – how often are these tests ordered for similar patient / different clinician | 1 | C3 |
| C4: Audit processes will be electronic, largely unobtrusive and will draw on ‘virtual registries’ that rely on data analytics. Feedback will be informed by the evidence on clinical decision making – incorporating passive ‘automated’ predictive analytics as well as peer to peer and expert feedback. | | |
| Electronic audit and feedback systems are in place (across all areas – inpatient, outpatient clinics, etc.) | 2 | C4 |
| Validated analytics for trends in patient-reported outcomes for performance feedback are established | 2 | C4 |
| Benchmarking and feedback approaches based on behaviour change and levers for change theory | 2 | C4 |
| Approaches that incorporate risk adjustment into benchmarking | 2 | C4 |
| Machine learning driven analytics incorporated into clinical audit | 2 | C4 |
| Coordinated and integrated clinical registry data seamlessly collected and made available to assess quality and safety of care for complex patients | 5 | C4 |
| Behavioural change (and culture change) with data analytics | 1 | C4 |
| C5: Data will be discussed within clinical teams so that clinicians can collectively assess the data and identify causes of variation and plan improvements. | | |
| All clinicians will receive regular data about their service delivery and outcomes from the previous quarter and with time trends analyses. These data will be discussed within clinical teams so that clinicians can collectively assess the data and identify causes of variation and plan improvements | 2 | C5 |
| Linkages with evidence based information on best practice | 2 | C5 |
| PRMs for patients with multiple conditions (e.g. cancer and COPD) are analysed, interpreted and reported in coordinated and meaningful ways | 5 | C5 |
| AI to capture qualitative elements e.g. care planning processes and guide how they relate to PRMs | 5 | C5 |
| Links between acute and primary health and procurement | 4 | C5 |
| Team based approaches need to be fed back | 2 | C5 |
| C6: Clinical research will be informed by data analytics, virtual registries, and big data. Efforts will be underway to secure wider data linkage to incorporate non-health sources. Clinician training will incorporate the use of analytics and address issues such as managing risk and uncertainty. | | |
| Partnerships with other jurisdictions | 4 | C6 |
| The application and interpretation of clinical analytics is an established topic in undergraduate courses and continuing professional education (with work placements in pillar organisations) | 6 | C6 |
| A greater proportion of clinical studies incorporating data generated by clinical analytics in results. | 6 | C6 |
| Larger and more complex datasets routinely used in clinical research (also available for education) | 6 | C6 |
| Embed research into practice; evaluation of business and outcomes; “learning organisation” | 6 | C6 |
| Hypothesis-generation and -testing based on clinical analytics featured in a multiple research institutes | 6 | C6 |
| Partnerships between pillars, Ministry of Health and academic partners established, attracting research funding and producing peer reviewed research. | 6 | C6 |
| Research agenda determined by system rather than researchers | 6 | C6 |
| Clinical analytics for research and education strategy developed for NSW Health (will include the work currently being developed by the Workforce Skills & Training Working Group) | 6 | C6 |
| Truly embedding research in health rather than just saying we do it | 6 | C6 |
| Linkages with non-NSW Health data to help guide practice e.g. GP data form Best Practice, Med Director, etc. or My-HR. Should help with looking at linger term outcomes of hospitalised patients | 2 | C6 |
| **Enablers** |  |  |
| Training – acceptance of feedback and how to incorporate the feedback into practice | 2 | C |
| Use large datasets to find interesting individual patients then study all the complexity – clinicians not interested as much in percentages | 2 | C |
| Medical / clinical staff training in analytics | 1 | C |
| Connected / integrated whole system datasets that provide meaningful insights into the patient journey across organisational boundaries and sectors to inform redesign and care | 3 | C |
| Using data that already exists for hypothesis generation | 3 | C |
| Training in communication skills for clinicians to incorporate use of clinical analytics in an authentic way to add to the clinical encounter | 6 | C |
| Embed into teaching examples clinicians are interested in solving real problems - then they will be drawn to the tools | 6 | C |
| Clinical researcher / fellow position | 6 | C |
| Identification of high value clinical questions | 6 | C |
| Education resources – HHL- HOOC | 6 | C |
| Linked pharmacy data – measuring adherence – PBS / ambulance/ inpatient outpatient My Health - access to this information that does not involve ethics | 5 | C |
| Embed registries within health rather than encouraging bespoke collections outside the system | 5 | C |
| Review LHD dashboards / pathology integration | 4 | C |
| Pathology and imaging appropriateness of ordering – right test on right patient at right time. Dashboard benchmarking comparisons and ML/AI on categories of testing on presenting/ diagnosis problems. Better standardisation of terminology and robust data linkage | 2 | C |
| Consistent integrated systems support the [broader policy making] framework across organisational | 4 | C |
| Incorporate evidence-based elements into data presentation e.g. reports may be presented quarterly but have more frequent data points so interpretations can be made along lines of special cause or common cause variation | 2 | C |
| Digital health literacy | 6 | C |
| Data capture/ extracted linked with clinical systems and admin systems. Models for interpretation of these (AI / machine learning] and other analytics | 4 | C |
| Research registries automated | 6 | C |
| **Patients’ Perspective** | | |
| P1: Patients in five years’ time will be firmly established as key informants in healthcare – providing data about their health status, experience and outcomes. | | |
| Patients are supported to participate in real time decision making | 1 | P1 |
| Patients are engaged and informed and report better outcomes and experiences of care | 2 | P1 |
| Recognition of the value of the ‘I statements’ (See separate document for those statements) | NA | P1 |
| P2: They will be engaged in monitoring their health using wearables that feed data into analytic repositories, and supported to manage their health issues – prompted by algorithm enabled alerts. | | |
| Personalised medicine approaches based on data analytics | 5 | P2 |
| Integrate patient generated data sets / wearables | 5 | P2 |
| Telemedicine support programs based on multi-morbid scoring system | 5 | P2 |
| P3: They will be assured that their data are secure, that the healthcare system will ‘find them’ and intervene at an early stage to avoid a crisis; and that they have the information and support they need. | | |
| Patients / family are able to also know their clinical data and how it is being used to guide treatment – family / patient able to escalate concern | 1 | P3 |
| Community input | 1 | P3 |
| **Managers’ Perspective** | | |
| M1: Managers, in five years’ time will be confident that monitoring and measurement systems are reliably and sensitively assessing healthcare services. They will be able to test models of reconfiguration and structural changes using data analytics.  Real time alerts regarding impending surges in demand in acute care areas such as emergency departments, operating theatres and critical care units will be used to manage workflows, staffing and bed management. | | |
| Streamlined system-level reporting or a reporting framework for variation in clinical care, patient journeys and outcomes | 3 | C2 /M1 |
| State-wide framework for clinical decision support systems – implementation and evaluation | 3 | M1 |
| Development of algorithms that draw on multiple data sources to guide policy priority setting | 3 | M1 |
| Analysis of coding and identification of gaps | 3 | M1 |
| Clarify who owns the data | 5 | M1 |
| Consistent data entry | 6 | M1 |
| Constant evaluation of algorithms and data sources | 6 | M1 |
| Data analytics algorithm to support administration in claims management | 4 | M1 |
| Capacity building in system – clinicians, managers, organisations (LHDs, pillars) – training, education including undergraduate. | 4 | M1 |
| M2: Service level and system managers will utilise data from clinical analytics alongside administrative and other data to guide policy development and improve performance. | | |
| Predictive analytics across multiple scenarios / patient groups for policy making and planning | 3 | M2 |
| Analytic networks or communities of practice are established across the state | 3 | M2 |
| Connect reports with levers for other purposes e.g. reports that lead to CPD that then informs any future revalidation need | 2 | M2 |
| Culture of constant testing and calibration of algorithms | 5 | M2 |
| Use data to evaluate models of care | 2 and 6 | M2 |
| Frameworks for clinical analytics used in assessment of integrated care | 5 | M2 |
| Programs to measure the impact that the knowledge generation and education is having on the health system | 6 | M2 |
| Inform public health intervention through early warning systems of clusters of presentations (DRG) | 3 | M2 |
| M3: There will be a robust mechanism and framework to identify, prioritise and support the introduction of system wide clinical analytic initiatives. | | |
| Protocols in place for data collection and real time correction or over-writes (needs to be simple / easy for clinicians inputting data) | 1 | M3 |
| Placing clinical analytics in a broader policy making framework | 3 | M3 |
| An efficient and effective working group is in place (defines standards; access to data etc.) | 4 | M3 |
| There is a robust mechanism and framework to identify, prioritise and support the introduction of system wide clinical analytic initiatives | 4 | M3 |
| A strategy for an enhanced data analytic approach to enhancing integrated care | 5 | M3 |
| Pharmacovigilance datasets linked to state based HIE to support public health responses – policy in pharma and device safety and funding | 3 | M3 |
| Within each LHD, form analysis units that bring together business, performance, academia, procurement to measurably improve health care | 4 | M3 |
| Establishment of an academy of clinicians involved in data analytics tool development | 2 | M3 |
| **Enablers** |  |  |
| Equity of resources across state | 6 | M3 |
| Central platform for loading new data sets – to be set up in a rapid / agile way | 5 | M3 |
| Need to embed a governance structure to underpin this – well recognised pathways; engage professional colleges, pillars, LHDs, primary health, research institutes | 4 | M3 |
| Recognise the centrality of informatics / analytics to deliver a constantly improving health system. Establish and invest in a NSW Health data Institute that brings together health, academia, industry, and community that is committed to [the above] this and work together in novel ways | 4 | M3 |
| LHD investment to innovate in this space – facilitated funding for analytics projects | 4 | M3 |
| Establish common methodology (remove analytic variation) | 3 | M3 |
| Established daily extractions of clinical data to the central lake | 3 | M3 |
| Establish agreed system-wide principles along lines of data for research; data for accountability; data for improvement | 3 | M3 |
| Infrastructure to enable it all | 1 | M3 |
| Build trust in the source of truth | 3 | M3 |
| State BI unit | 3 | M3 |
| Data governance | 3 | M3 |
| Owned by clinicians | 3 | M3 |
| Co-develop continuous testing and calibration of algorithms | 3 | M3 |
| Capitalise on existing high value data collections to show how real time analytics can inform safety and quality issues e.g. maternity | 3 | M3 |
| Use clinical variation to trial funding models that support best care practice | 3 | M3 |
| Equity of access to resources for D&A across whole state | 3 | M3 |
| Sydney Triage to Admission tool – pilot currently underway | 1 | M3 |
| Data used to predict patient journey – front load logistic planning | 1 | M3 |
| Analysis of existing sources and identification of gaps (access, skills/ training) | 5 | M3 |
| How is it actioned – directing to lowest level of safe care e.g. HITH | 1 | M3 |
| What is the toolset that NSW Health will invest in? | 4 | M3 |
| Designed and evaluated a strategy to provide data and feedback to clinicians | 2 | M3 |
| Consolidate resources | 3 | M3 |
| Leader in internal benchmarking systems (local) | 2 | M3 |

**Appendix 3: Barriers and enablers identified in workshop**

| **Enablers** | **Barriers** |
| --- | --- |
| **Technology exists**  **Millennials and paperless**  **High quality care**  **Targeted earlier**  **Information for patient** | Traditional model doesn’t work  Lack of standards  Workforce team silos |
| Natural Language Processing  View data as a by-product of clinical care  Support of skilled analytic workforce  Support for data management across the system (make a priority)  Good system workflows must be contextual to the care delivered  System wide master data management  Clarity on data ownership sharing principles  Risk adjustment  education | Clinician time access logins  Lack of integration between systems and data sources  Knowing where to go  Inconsistent tools  Fragmentation of data locations for one individual patient  Resources – human, technical / tools  Security and governance – inconsistency, lack of clarity about how to access in well governed way  Quality of data available as a consequence of data collection and attitude and system design |
| Well-designed reports / tools  Co-design approach – clinicians and data people  Trust  Diagnosis / investigations focus (dx/ix)  Communication  Culture of quality improvement  Mechanisms / tools / resources to enable deep dive into data  80:20 rule  Start with standard tweakable but not breakable | Poorly designed reports / data supply  Punitive use of info with clinicians  Use for purposes other than intended e.g. unfair comparisons |
| Unique identifiers across sector  Public perception – articulating benefits of My Health  Community expectations  Governance framework / security  Communication technologies / real time feeds  Broadband  Cloud computing analysis  Reliable technological infrastructure | Legislative framework  Community education  Access to data silos |
| Codesign between clinician management data people patients  Start with simple standard design and build from there 80:20 rule  Trust and communicate | Misuse of data e.g. unfair comparisons  Fragmented data sources  Required effort skill to bring together  Lack of integration between systems for data   1. Capture   Reporting |
| Good will (enthusiastic clinicians / partners)  Policy drive in changing practice / provision healthcare  Consumer expectation  Value based care  Increase efficiency: health budget  Increase in technology / paperless  Health internet infrastructure – harmony  Current worldwide models to understand lessons learned  Millennials utilise time IT models  Opportunity for AI / IT to implement | Lack of technology / standard  Different technology models within LHDs  Lack of clinical champions to lead analytics  Trust  Education  Workforce limitation – time  User acceptability  Resources  Relying on traditional implementation model  Current infrastructure is limited  Lack of investment in infrastructure |
| Terminology available  Technology exists to bring data together  Capacity available | Lack of standard definitions, data sets  Terminology not implemented  Tools available to extract data in real time  Hybrid record  Lag in time of results available  Link  Lack of knowledge in workforce and tech  Cerner doesn’t work |
| Technology   - triangulation (business, clinical, workforce, financial) - consistent platform - digital solution (not just add on) - already capturing quant and qual   Process   - strong clinical and corporate governance - streamlined process framework – clinical to state   People   - skill people - network – at site level but connected to central site | Technology   - silo - no single visibility   Process   - silo process and people - no single process for system response - different systems for different data   People   - trust |

**Appendix 4: Online Survey**

Total number of participants: 20

| **Question 1** | **% Accept** | **% Do Not Accept** | **% Modification** |
| --- | --- | --- | --- |
| C1: Clinicians will, as a matter of course, use patient reported measures (gathered via patients’ phones and tablets), incorporated into other diagnostic and prognostic markers and inputs. | 64 | 5 | 31 |

| **Question 2** | **% Accept** | **% Do Not Accept** | **% Modification** |
| --- | --- | --- | --- |
| C2: Algorithms will assess patterns, patient trajectories and risk strata for patients and this information will be considered in the consultation. | 59 | 14 | 27 |

| **Question 3** | **% Accept** | **% Do Not Accept** | **% Modification** |
| --- | --- | --- | --- |
| C3: Very targeted and well validated alerts will highlight risk and safety issues. | 86 | 0 | 14 |

| **Question 4** | **% Accept** | **% Do Not Accept** | **% Modification** |
| --- | --- | --- | --- |
| C4: Aggregated, time-series data will be collected unobtrusively through eMR and other routine clinical tasks. | 82 | 5 | 13 |

| **Question 5** | **% Accept** | **% Do Not Accept** | **% Modification** |
| --- | --- | --- | --- |
| C5: Clinicians will have access to timely information that allows for reflective practice, highlighting whether their results point to unwarranted clinical variation – that is, when “patient care that differs in ways that are not a direct and proportionate response to available evidence; or to the healthcare needs and informed choices of patients”. | 73 | 0 | 27 |

| **Question 6** | **% Accept** | **% Do Not Accept** | **% Modification** |
| --- | --- | --- | --- |
| C6: Information will be automatically generated on-Concordance with evidence-based practice-Diffusion rates of new technologies-Risk adjusted patient outcomes-Benchmarking comparisons-Time-series and patient trajectories | 62 | 19 | 19 |

| **Question 7** | **% Accept** | **% Do Not Accept** | **% Modification** |
| --- | --- | --- | --- |
| C7: Machine learning processes, deployed to discern novel patterns in complex and large datasets, will inform the development of algorithms and highly specific alerts. | 63 | 14 | 23 |

| **Question 8** | **% Accept** | **% Do Not Accept** | **% Modification** |
| --- | --- | --- | --- |
| C8: Audit processes will be electronic, largely unobtrusive and will draw on ‘virtual registries’ that rely on data analytics. | 71 | 10 | 19 |

| **Question 9** | **% Accept** | **% Do Not Accept** | **% Modification** |
| --- | --- | --- | --- |
| C9: Feedback will be informed by the evidence on clinical decision making – incorporating passive ‘automated’ predictive analytics as well as peer to peer and expert feedback. | 82 | 9 | 9 |

| **Question 10** | **% Accept** | **% Do Not Accept** | **% Modification** |
| --- | --- | --- | --- |
| C10: Data will be discussed within clinical teams so that clinicians can collectively assess the data and identify causes of variation and plan improvements. | 86 | 0 | 14 |

| **Question 11** | **% Accept** | **% Do Not Accept** | **% Modification** |
| --- | --- | --- | --- |
| C11: Clinical research will be informed by data analytics, virtual registries, and big data. | 68 | 9 | 23 |

| **Question 12** | **% Accept** | **% Do Not Accept** | **% Modification** |
| --- | --- | --- | --- |
| C12: Efforts will be underway to secure wider data linkage to incorporate non-health sources | 86 | 5 | 9 |

| **Question 13** | **% Accept** | **% Do Not Accept** | **% Modification** |
| --- | --- | --- | --- |
| C13: Clinician training will incorporate the use of analytics and address issues such as managing risk and uncertainty. | 91 | 0 | 9 |

| **Question 14** | **% Accept** | **% Do Not Accept** | **% Modification** |
| --- | --- | --- | --- |
| P1: Patients will be assured that:-their data are secure,-that the healthcare system will ‘find them’ and intervene at an early stage to avoid a crisis;-they have the information and support they need. | 55 | 9 | 36 |

| **Question 15** | **% Accept** | **% Do Not Accept** | **% Modification** |
| --- | --- | --- | --- |
| P2: Patients will be firmly established as key informants in healthcare – providing data about their health status, experience and outcomes. | 91 | 0 | 9 |

| **Question 16** | **% Accept** | **% Do Not Accept** | **% Modification** |
| --- | --- | --- | --- |
| P3: Patients will be engaged in monitoring their health using wearables that feed data into analytic repositories. | 68 | 9 | 23 |

| **Question 17** | **% Accept** | **% Do Not Accept** | **% Modification** |
| --- | --- | --- | --- |
| P4: Patients will be supported to manage their health issues. | 77 | 9 | 14 |

| **Question 18** | **% Accept** | **% Do Not Accept** | **% Modification** |
| --- | --- | --- | --- |
| P5: Self-management will be prompted by algorithm enabled alerts. | 73 | 14 | 14 |

| **Question 19** | **% Accept** | **% Do Not Accept** | **% Modification** |
| --- | --- | --- | --- |
| M1: Managers will be confident that monitoring and measurement systems are reliably and sensitively assessing healthcare services. | 86 | 0 | 14 |

| **Question 20** | **% Accept** | **% Do Not Accept** | **% Modification** |
| --- | --- | --- | --- |
| M2: Managers will be able to test models of reconfiguration and structural changes using data analytics. | 86 | 5 | 9 |

| **Question 21** | **% Accept** | **% Do Not Accept** | **% Modification** |
| --- | --- | --- | --- |
| M3: Real time alerts regarding impending surges in demand in acute care areas such as emergency departments, operating theatres and critical care units will be used to manage workflows, staffing and bed management. | 90 | 5 | 5 |

| **Question 22** | **% Accept** | **% Do Not Accept** | **% Modification** |
| --- | --- | --- | --- |
| M4: Service level and system managers will utilise data from clinical analytics alongside administrative and other data to guide policy development and improve performance. | 95 | 0 | 5 |

| **Question 23** | **% Accept** | **% Do Not Accept** | **% Modification** |
| --- | --- | --- | --- |
| M5: There will be a robust mechanism and framework to identify, prioritise and support the introduction of system wide clinical analytic initiatives. | 100 | 0 | 0 |

**Appendix 5: Final Delphi**

Total Number of Participants: 16

Options provided to participants for each statement:

- I accept this statement as modified
- I do not accept this statement
- I propose further modification of this statement

| **Final Statement** | **Final Result** |
| --- | --- |
| C1: Patient reported measures will be part of routine care. They will be used for diagnosis, prognosis and clinical decision making. | Accept: 100% |
| C2: Clinically validated algorithms will assess case histories, diagnoses and risk profiles. They will facilitate safe and effective clinical care. | Accept: 100% |
| C5: Clinicians will have access to relevant and timely information that highlights any unwarranted clinical variation and supports reflective and current best practice. | Accept: 92%  Further modification: 8% |
| C6: Information will be available at the point of care on: concordance of clinicians' care with evidence-based practice; risk adjusted patient outcomes; benchmarking and peer comparisons; time-series and patient trajectories. | Accept: 93%  Do not accept: 7% |
| C7: Advanced analytics or AI approaches are deployed to discern novel patterns in complex and large datasets and guide the development of algorithms. | Accept: 100% |
| C8: Analytics driven clinical audit processes will draw on "virtual registries" to personalise learning | Accept: 86%  Further modification: 14% |
| C11: Clinical research will be informed by timely and efficient access to linked data, big data, "virtual registries" and analytics. | Accept: 100% |
| P1: Patients will be assured that their data are appropriately secure and used to support clinical care and quality improvement. | Accept: 100% |
| P3: Patients who chose to, will be engaged in monitoring their health using technologies that can communicate with information systems. | Accept: 86%  Further modification: 14% |
| P4: Patients will be enabled and supported to access their own data and to use it to manage their health. | Accept: 93%  Do not accept: 7% |
| P5: With patient consent, self-management will be prompted by algorithm enabled alerts | Accept: 100% |
